# Proteomic Analysis of Highly Prevalent Amyloid A Amyloidosis Endemic to Endangered Island Foxes

**DOI:** 10.1371/journal.pone.0113765

**Published:** 2014-11-26

**Authors:** Patricia M. Gaffney, Denise M. Imai, Deana L. Clifford, Majid Ghassemian, Roman Sasik, Aaron N. Chang, Timothy D. O’Brien, Judith Coppinger, Margarita Trejo, Eliezer Masliah, Linda Munson, Christina Sigurdson

**Affiliations:** 1 Departments of Pathology and Medicine, University of California San Diego, La Jolla, California, United States of America; 2 Department of Pathology, Immunology, and Microbiology, University of California Davis, Davis, California, United States of America; 3 Wildlife Investigations Laboratory, California Department of Fish and Wildlife, Rancho Cordova, California, United States of America; 4 Department of Veterinary Medicine and Epidemiology, University of California Davis, Davis, California, United States of America; 5 Department of Chemistry and Biochemistry, University of California San Diego, La Jolla, California, United States of America; 6 Center for Computational Biology, Institute for Genomic Medicine, University of California San Diego, La Jolla, California, United States of America; 7 Veterinary Population Medicine Department, Veterinary Diagnostic Laboratory, University of Minnesota, St. Paul, Minnesota, United States of America; 8 Departments of Pathology and Neuroscience, University of California San Diego, La Jolla, California, United States of America; University of Melbourne, Australia

## Abstract

Amyloid A (AA) amyloidosis is a debilitating, often fatal, systemic amyloid disease associated with chronic inflammation and persistently elevated serum amyloid A (SAA). Elevated SAA is necessary but not sufficient to cause disease and the risk factors for AA amyloidosis remain poorly understood. Here we identify an extraordinarily high prevalence of AA amyloidosis (34%) in a genetically isolated population of island foxes (*Urocyon littoralis)* with concurrent chronic inflammatory diseases. Amyloid deposits were most common in kidney (76%), spleen (58%), oral cavity (45%), and vasculature (44%) and were composed of unbranching, 10 nm in diameter fibrils. Peptide sequencing by mass spectrometry revealed that SAA peptides were dominant in amyloid-laden kidney, together with high levels of apolipoprotein E, apolipoprotein A-IV, fibrinogen-α chain, and complement C3 and C4 (false discovery rate ≤0.05). Reassembled peptide sequences showed island fox SAA as an 111 amino acid protein, most similar to dog and artic fox, with 5 unique amino acid variants among carnivores. SAA peptides extended to the last two C-terminal amino acids in 5 of 9 samples, indicating that near full length SAA was often present in amyloid aggregates. These studies define a remarkably prevalent AA amyloidosis in island foxes with widespread systemic amyloid deposition, a unique SAA sequence, and the co-occurrence of AA with apolipoproteins.

## Introduction

Amyloid A (AA) amyloidosis is among the most common systemic amyloidoses in humans and animals and follows recurring episodes of inflammation and concurrently elevated serum amyloid A (SAA) protein levels [1]–[4]. SAA is synthesized primarily in hepatocytes in response to the pro-inflammatory cytokines IL-1, IL-6, and TNF-α, which activate STAT3, NF-κB p65, and p300 complex formation at the SAA promoter and induce SAA transcription [5]. Persistently high SAA levels can trigger extracellular AA fibril formation [6], progressive amyloid deposition, and eventually organ dysfunction [7], most frequently renal failure [8].

SAA is a highly conserved protein composed of 104 to 112 amino acids [9] and human SAA is arranged in a four helix bundle that assembles into a hexamer [10]. The SAA hexamer binds high density lipoproteins (HDL) through at least two binding sites [10], [11]. SAA also contains binding sites for glycosaminoglycans including heparan sulfate, which has been implicated in promoting SAA aggregation [10], [12], [13].

The mechanism whereby soluble, alpha-helical SAA converts into insoluble, β-sheet-rich fibrils is incompletely understood. SAA amino acids 1–15 have been shown to be highly aggregation prone in vitro [14], and prediction algorithms used to identify aggregation prone peptide segments, Zipper DB and TANGO, indicate that residues 2–8 and 52–59 are amyloidogenic [10], [15], [16]. The N-terminal 1-76-residue SAA fragment is often predominant in amyloid aggregates, indicating protease cleavage can occur [17]–[19].

Despite the high frequency of chronic inflammatory disease, most individuals do not develop AA amyloidosis. Risk factors for AA amyloidosis among individuals with chronic inflammatory diseases include certain SAA allelic variations or SNPs, for example, SAA1.1 and SAA1.3 alleles in Caucasian and Japanese populations, respectively, or a SNP at −13(T) in the 5′UTR [20]–[23]. A point mutation in the SAA promoter was found in highly inbred captive cheetahs that have an extraordinarily high prevalence of AA amyloidosis (70%) [24]. This point mutation increased SAA transcription under inflammatory conditions in vitro [24], [25].

Here we used shotgun proteomics to identify AA as the cause of a highly prevalent systemic amyloidosis of island foxes, a genetically-isolated, endangered species from the California Channel Islands [26]. Shotgun proteomics has recently emerged as a technique that not only resolves the amyloidogenic protein but also identifies amyloid-associated proteins, such as serum amyloid P, apolipoprotein E and apolipoprotein A-IV [27], [28]. We have assembled the island fox AA sequence as well as variants, identified amyloid-associated proteins, and characterized the widespread organ tropism of the amyloid, which tracks along basement membranes in diverse tissue types. These findings, particularly the unique AA sequence in a population with limited genetic variability, suggest an underlying genetic contribution to AA amyloidosis that may inform on the complex interplay between inflammatory mediators and AA fibrillogenesis.

## Materials and Methods

### Ethics statement

Tissues used in this study were collected from island fox carcasses recovered from the California Channel islands (Santa Cruz, Santa Rosa, San Miguel, San Clemente, San Nicolas, and Santa Catalina). The island fox is a state listed endangered species of California, USA, and subspecies from Santa Cruz (*Urocyon littoralis santacruzae*), Santa Rosa (*U. l. santarosea*), San Miguel (*U. l. littoralis*), and Santa Catalina (*U. l. catalinae*) islands are also federally listed endangered species [29], [30]. The United States Fish and Wildlife Service (TE-744878-14) and California Department of Fish and Wildlife [Memorandum of Understanding, Institute for Wildlife Studies, Dave Garcelon (SCP-00932)] permits authorize carcass recovery and use from all island fox subspecies. Lands were accessed with permission by employees or designated contractors of the land owner. Lands accessed were protected by the National Park Service (Santa Cruz, Santa Rosa, San Miguel) and The Nature Conservancy (Santa Cruz), owned and operated by the United States Navy (San Clemente, San Nicolas) or publicly managed by the Catalina Island Conservancy (Santa Catalina).

### Animals and sample collection

Island foxes (n = 321) that died between 1987 and 2010 were necropsied for disease surveillance, and included all six subspecies: Santa Cruz (*Urocyon littoralis santacruzae*), Santa Rosa (*U. l. santarosea*), San Miguel (*U. l. littoralis*), San Clemente (*U. l. clementae*), Santa Catalina (*U. l. catalinae*) and San Nicolas (*U. l. dickeyi*). Demographic characteristics [age, sex, year of death, captivity status, and concurrent diseases] were recorded for each animal. Captivity status was defined as either free-ranging or captive. Captive animals included foxes hospitalized for illness, and those housed in captive breeding pens or in mainland zoos. Chronic inflammatory diseases included otitis externa from *Otodectes sp.* ear mites, intestinal granulomas associated with *Spirocerca sp.* nematodes, abscesses or cellulitis from trauma or bacterial infection, as well as chronic nephritis, pneumonia, hepatitis, enteritis, endocarditis, and arthritis. The X^2^ test was used to assess the association between demographic characteristics and amyloidosis; p≤0.05 was considered significant. Systematic post-mortem examinations were performed and tissues were frozen and fixed in 10% buffered formalin. Formalin-fixed tissues were paraffin-embedded and 5 µm sections were mounted on glass slides and stained with hematoxylin and eosin as well as Congo red. Necropsy databases from the University of California, Davis Veterinary Medical Teaching Hospital (VMTH), California Animal Health and Food Safety Laboratories (CAHFS), and Southeastern Cooperative Wildlife Disease Study (SCWDS) were queried to determine if amyloidosis had been previously diagnosed in mainland gray foxes (n = 410) (*Urocyon cinereoargenteus*), the closest genetic relative to the island fox.

### Immunohistochemistry for SAA

Immunohistochemistry for SAA was performed using the avidin-biotin-peroxidase method. Endogenous peroxidase was quenched with 3% hydrogen peroxide. Tissues were then blocked, sequentially incubated in polyclonal rabbit anti-canine AA antibody [31], [32], biotinylated goat anti-rabbit secondary, and streptavidin horse-radish peroxidase, and then visualized using a 3,3′-diaminobenzidine substrate. Positive controls included a Shar Pei dog kidney and a duck liver with presumed AA amyloid (based on concurrent inflammatory disease and a predisposition for developing AA amyloidosis). Negative control island fox tissues lacking congophilic deposits, as well as tissues containing known non-AA amyloids (islet amyloid polypeptide and prion protein), were included to test for specificity. Additionally, rabbit IgG isotype substitution of the primary antibody was used on amyloid positive fox tissues as an additional control for specificity.

### Electron microscopy of kidney

Island fox kidneys with or without AA amyloid (confirmed by Congo red stain) were fixed in Karnofsky’s solution, post-fixed in osmium tetroxide, embedded in Epon resin, sectioned at 60 nm onto nickel grids, and negatively stained with saturated uranyl acetate in 50% ethanol and bismuth sub-nitrate solution. Grids were analyzed with a Zeiss EM10 electron microscope.

### Insoluble protein preparation and immunoblotting

Cortex and medulla (50–150 mg) from 7 kidneys with more than 50% amyloid in either region and 7 kidneys with no microscopically visible amyloid were homogenized in 10 volumes (w/v) of PBS using a Beadbeater homogenizer (Biospec, Bartlesville, OK). Homogenates were lysed in Tris-HCl with 1% sarcosyl (final) at 37°C for 30 minutes with agitation, and centrifuged for 30 minutes at 18,000 g. The insoluble pellet fraction was solubilized in 100 mM Tris HCl (pH 8.0) containing 10 M urea at 37°C for 30 minutes with agitation, and then diluted with 100 mM Tris-HCl to 4 M urea (final). Samples were then electrophoresed through a 10% Bis-Tris, SDS-polyacrylamide gel (Life Technologies, Grand Island, NY) and visualized with Coomassie blue. A duplicate gel was transferred to a nitrocellulose membrane and immunoblotted using a polyclonal rabbit anti-canine AA antibody [32] and a goat anti-rabbit HRP conjugated secondary antibody. Membranes were developed using Pierce SuperSignal West Dura chemiluminescent substrate (Thermo Scientific, West Palm Beach, FL) and visualized on a Fuji 4000 chemiluminescent imager. Omission of the primary and use of the secondary antibody only was used as a negative control.

### Mass spectrometry and LC-MS/MS

Protein lysates in 4 M urea from a subset of 7 amyloid positive kidneys (5 cortex, 6 medulla) and 3 amyloid negative kidneys (3 cortex, 2 medulla) (amyloid status of cortex and medulla defined by Congo red positivity) were used for tryptic digestions. Samples were reduced in 5 mM dithiothreitol (DTT) (final) for 20 minutes at room temperature, alkylated in 10 mM iodoacetamide for 15 minutes at room temperature, and digested in 1.0 µg/µl mass spectrometry grade trypsin-gold (Promega, Madison, WI) and 2 mM CaCl_2_ with agitation for 48 hours at 37°C. Samples were centrifuged at 16,000 g for 30 minutes at 4°C and peptides were extracted and desalted using Aspire RP30 desalting columns (Thermo Scientific).

Trypsin-digested peptides were analyzed by high pressure liquid chromatography electrospray ionization tandem mass spectrometry (HPLC-ESI-MS/MS). The nanospray ionization experiments were performed using a TripleTof 5600 hybrid mass spectrometer (ABSCIEX, Framingham, MA) interfaced with nano-scale reversed-phase HPLC (Eksigent technologies, San Francisco CA) using a 10 cm–100 micron ID glass capillary packed with 5 µm C18 Zorbax beads (Agilent Technologies, Santa Clara, CA). Peptides were eluted from the C18 column into the mass spectrometer using a linear gradient (5–60%) of acetonitrile (ACN) at a flow rate of 250 µl/min for 1 h. The ACN gradient was created with Buffer A (98% H_2_O, 2% ACN, 0.2% formic acid, and 0.005% trifluoroacetic acid (TFA)) and Buffer B (100% ACN, 0.2% formic acid, and 0.005% TFA). MS/MS data were obtained in a data-dependent manner, acquiring MS1 data for 250 ms at m/z of 400 to 1250 Da followed by 50 MS/MS events for 25 ms accumulation time for each event. Criteria for independent data acquisition (IDA) were 200 count threshold, charge state of +2 to +4, with 4 seconds of exclusion time.

The acquired spectra were analyzed using the PARAGON Algorithm [33] and Protein Pilot 4.0 (ABSCIEX) for peptide identifications querying the *Canis lupus familiaris* proteome. The canine proteome database was supplemented with the predicted SAA protein sequences from island and gray fox SAA cDNA sequences and from published SAA and serum amyloid P (SAP) protein sequences from numerous species (human, mouse, cat, horse, cow, mink, arctic fox, cheetah, ferret, mallard, flamingo, and Syrian hamster). Primers used to amplify fox SAA cDNA were 5′ CACCATGAAGCTTTTCCCGG 3′ (forward) and 5′ TCAGTACTTGTCAGGCAGGC 3′ (reverse). Spectral counts were normalized against the whole protein complex using published methods [34] and reported as percent total insoluble protein. Only peptides identified with≥95% confidence were considered. Tryptic peptide alignments were done using ClustalOmega (Copyright EMBL-EBI 2013) and variants were identified based on identification of one or more amino acids at a single location in the protein.

For differential protein abundance analysis, normalized spectral counts, summed for each protein, were transformed with a function *x →* log_2_(1+*x*) to stabilize variance. Proteins were sorted according to their *q-*value, the smallest false discovery rate (FDR) at which the protein is called significant [35]. An FDR value of α is the expected fraction of false positives among all proteins with *q* ≤ α. FDR was evaluated using Significance Analysis of Microarrays and its official implementation *samr* [36]. Heatmaps of transformed protein abundance for all proteins with *q*-value ≤0.05 were created using in-house hierarchical clustering software, which implements Ward clustering. The colors qualitatively correspond to fold changes *f* with respect to a reference level defined for each protein as the mean of the two groups. Maximum color saturation is used whenever |*f*| ≥2.

### Amyloidogenicity prediction

Protein sequence and isoforms were assessed for amyloid-prone regions using three algorithms: Tango [15], Waltz [37], and Zyggregator [38].

## Results

### Prevalence of amyloidosis

We identified 109 of 321 necropsied island foxes (34%) with systemic amyloidosis. There were nearly equal proportions of males and females (males: 32%; females: 36%), but 4 times more adults than juveniles with amyloidosis (adults: 56%; juveniles: 14%) (Table 1). The mean number of cases of amyloidosis per year was 38% [range: 20–67%; standard deviation: 15], and cases did not significantly increase over time. Concurrent chronic inflammatory diseases, including otitis externa from ear mites as well as intestinal granulomas from nematodes, were widespread in the study population [88% (283/321)]; however, chronic inflammation was not significantly associated with amyloidosis (Table 1), suggesting additional factors influenced the development of amyloid. These findings are reminiscent of humans with rheumatoid arthritis, in which 10 to 30% have AA amyloidosis [39]–[41]. Captive housing was significantly associated with amyloidosis (p≤0.0001) as the proportion of cases of amyloidosis was higher in captive than free-ranging foxes (59% versus 27%, respectively). Amyloidosis occurred concurrently with neoplasia in a significant number of cases (75%) compared to non-cases (31%) (p≤0.0001). There were no diagnoses of amyloidosis identified in gray foxes (n = 401), the closest genetic relative of the island fox, in diagnostic laboratory databases from California and eastern U.S. states.

**Table 1:** Demographics of island foxes with systemic amyloidosis between 1987 and 2010.

Characteristic	Descriptor	Proportion with systemic amyloidosis *	Percent
*Urocyon littoralis*	All subspecies	109/321	34
	Juvenile/Young adult	22/161	14
Age	Adult/Geriatric	86/154†	56
	Unknown	1/6	17
	Female	57/157	36
Sex	Male	52/163	32
	Unknown	0/1	0
Chronic inflammation	Yes	91/283	32
	No	18/38	47
	Captivity	39/66‡	59
Captivity status	Free-ranging	69/253	27
	Unknown	1/2	50
Neoplasia	Yes	18/24§	75
	No	91/297	31

^*^# foxes with systemic amyoidosis/total # foxes necropsied (%).

† p≤0.0001, Yates X^2^ = 60.3.

‡ p≤0.0001, Yates X^2^ = 22.3.

§ p≤0.0001, Yates X^2^ = 17.6.

### Widespread distribution of amyloid among organs

**Figure 1:**
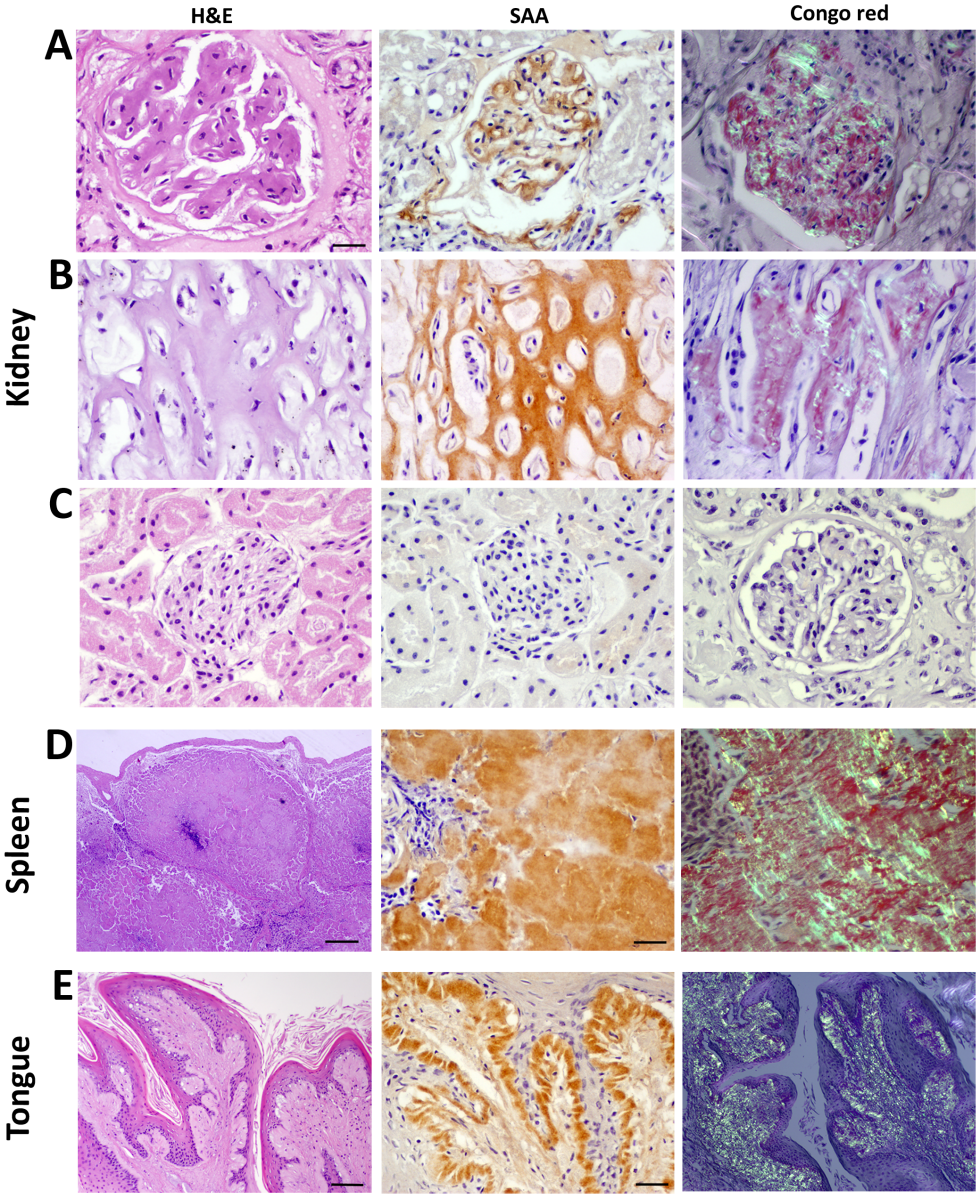
Histochemical and immunohistochemical staining of island fox amyloid. **A:** Amyloid markedly thickens the capillary loops of the glomerular tuft, labels with anti-AA antibody and shows birefringence when stained by Congo red. **B:** The renal medullary interstitium is expanded and tubules compressed by congophilic AA amyloid. **C:** Glomeruli and interstitium without amyloid show no immunoreactivity to canine AA antibody and are not congophilic. **D:** In the spleen, amyloid deposits in extensive, expansile nodules, replacing the white pulp and red pulp, and compressing smooth muscle trabeculae. **E:** Glossal papillae show hyperplasia and hyperkeratosis and a diffuse, thick submucosal band of amyloid that extends along the basement membrane. Scale bars = 50 µm [A, B, C, D (middle and right), E (middle)]; 200 µm [E (left and right)]; and 500 µm [D (right)].

We assessed the distribution of the amyloid systemically, and found that amyloid deposits were remarkably widespread, most commonly found in kidney, spleen, oral cavity, vessels, and heart (Table 2). Some foxes also had amyloid in adrenal gland, skin, paw pad, intestine, lung, thyroid gland, esophagus, ear canal, liver, lymph node and ovary. Amyloid deposits in all organs stained with Congo red and appeared birefringent under polarized light (Figure 1).

**Table 2:** Organs affected in cases of systemic amyloidosis.

Organ*	Proportion with amyloid†	Percent
Kidney	80/105	76
Spleen	57/99	58
Oral cavity	46/102	45
Vasculature	48/110	44
Heart	38/107	36
Adrenal gland	28/99	28
Skin/Paw pad	24/106	23
Gastrointestinal	22/104	21
Lung	19/105	18
Thyroid gland	15/95	16
Esophagus	14/99	14
Ear canal	11/97	11
Liver	9/105	9
Lymph node	7/93	8
Ovary	3/48	6

*Organs with rare amyloid were salivary gland and trachea (n = 2) and urinary bladder, gall bladder, pancreas, uterus, eye, and a joint (n = 1).

† # foxes with amyloid in an organ/total # organs examined in foxes with amyloid.

Renal amyloid deposited in the glomerulus, medullary interstitium, and vessels. Glomerular amyloid deposits ranged from segmental to global expansion of the entire tuft, and from affecting few to most glomeruli (Figure 1A). The medullary deposits ranged from mild to marked expansion of the interstitium (Figure 1B), sometimes with tubular atrophy and papillary necrosis. Amyloid frequently deposited along the internal elastic membranes of arcuate and medullary vessels as well as basement membranes of proximal and distal tubules. Amyloid also markedly expanded the vascular and tubular walls.

**Figure 2:**
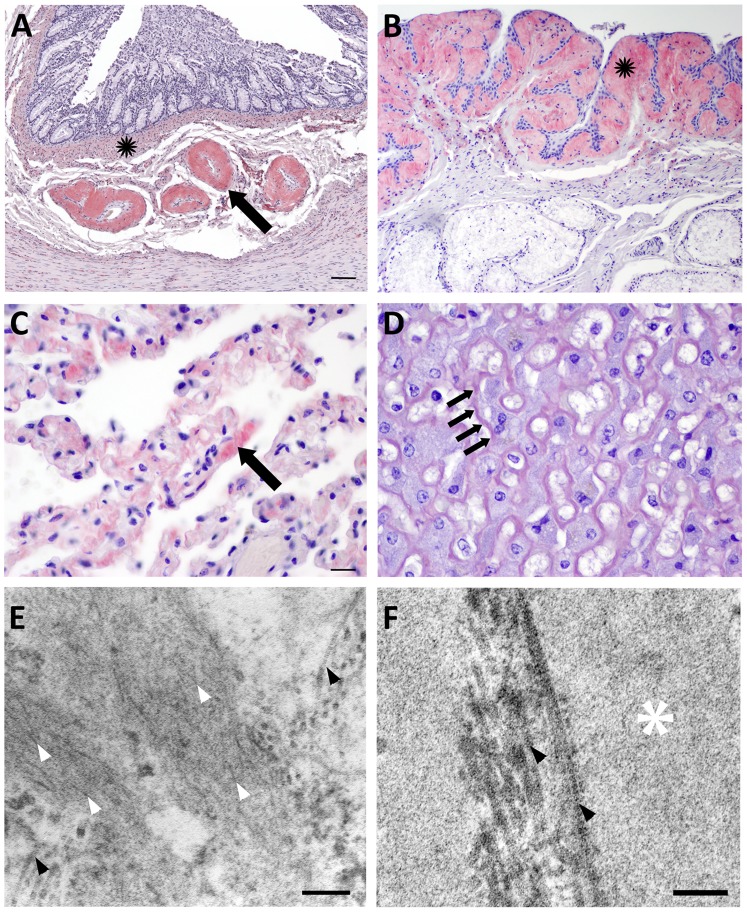
Congo red staining and electron microscopy of amyloid. **A:** Amyloid markedly expands colonic submucosal arteries (arrow) and accumulates diffusely throughout the muscularis mucosae (black star). **B:** The epiglottis mucosa is undulating and hyperplastic and is expanded by submucosal amyloid (black star). **C:** Amyloid deposits thicken the capillary walls in the alveolar septae of the lung (arrow). **D:** Amyloid widens the Space of Disse in the liver (arrows) disrupting hepatic plates and isolating hepatocytes. **E:** The renal medullary interstitium from an amyloid positive fox shows long, thin, non-branching fibrils approximately 10 nm diameter (white arrowheads) arranged haphazardly amongst thicker bundles of collagen (black arrowheads). F: The renal medulla from an amyloid negative fox contains basement membrane (white star) and collagen (black arrowheads), but no fibrils. Scale bars = 200 µm (A, B); 50 µm (C, D); 200 nm (E); and 50 nm (F).

Splenic amyloid was most frequently follicular and occasionally present as nodular aggregates, encircling afferent arterioles and lymphoid follicles with occasional replacement of white and red pulp (Figure 1D). Amyloid also deposited in the smooth muscle trabeculae, arteries, and capsule. Nodular amyloid deposits also expanded and deformed the tongue, pharynx, larynx and epiglottis, sometimes causing esophageal or tracheal obstruction. These tissues histologically showed a dense band of amyloid along the epithelial basement membrane as well as vascular amyloid and epithelial hyperplasia (Figure 1E, 2B).

Vascular amyloid deposits expanded the arteries and veins of every organ except brain, extending along the internal elastic membrane and/or expanding the vessel wall, sometimes occluding the lumen (Figure 2A). Cardiac amyloid localized to valves, myocardium, and coronary arteries. Pulmonary amyloid thickened the alveolar septae (Figure 2C) and hepatic amyloid deposited in the Space of Disse (Figure 2D). Similar to oropharyngeal tissue, amyloid tracked along the basement membrane in a dense, submucosal band in many tissues including the intestines, skin, paw pads, hair follicles, esophagus, and trachea.

### Amyloid protein identification and ultrastructure

Most systemic amyloid diseases in animals have been classified as AA amyloidoses. To identify the fox amyloid, we performed immunohistochemical stains using a polyclonal anti-canine AA antibody with known cross-species immunoreactivity [24], [31], [32]. Congophilic, birefringent amyloid deposits in kidney, spleen, and tongue (Figure 1A, B, D, E) and all other affected organs (not shown) were labeled by the anti-canine AA antibody, but not by an isotype control antibody. Identical tissues without amyloid showed no immunoreactivity (Figure 1C). To confirm specificity of the antibody, we tested tissues containing other amyloids, including islet amyloid polypeptide and prion protein, which did not bind the AA-antibody.

To characterize the amyloid ultrastructurally, we examined congophilic deposits from renal medulla by electron microscopy and found randomly-oriented thin bundles of non-branching fibrils. Fibrils were approximately 7–10 nm in diameter (Figure 2E), consistent with other protein fibrils found in amyloid [42]. No fibrils were evident in renal medulla that lacked congophilic deposits (Figure 2F).

### Biochemical properties of the AA amyloid

**Figure 3:**
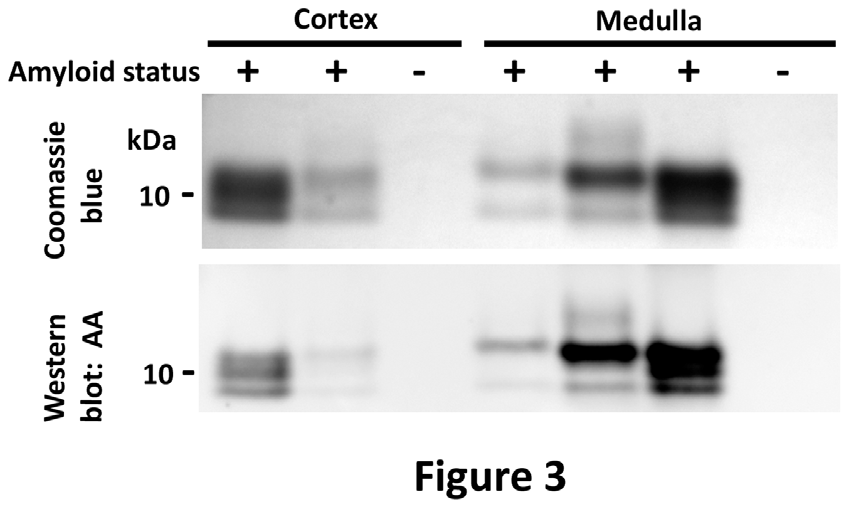
SDS-PAGE and western blot of the dominant insoluble protein in amyloid-laden kidneys. Coomassie blue stain of insoluble proteins from the renal cortex and medulla show a dominant band at 12 kDa and one or two lower molecular weight bands at 10 and 8 kDa, consistent with full length and fragments of SAA. An immunoblot shows that 12 kDa and lower molecular weight bands react with anti-canine AA antibody.

We next tested the biochemical properties of the insoluble protein fraction of amyloid-laden and control fox kidneys (n = 14 samples of each). Tissue homogenates from renal cortex or medullary samples in Tris-HCl with 1% sarcosyl were centrifuged, and pelleted proteins were solubilized in Tris-HCl with 10 M urea. Proteins diluted to 4M urea were electrophoresed by SDS-PAGE and the gel was stained with Coomassie blue. Interestingly, the stained gel revealed a dominant 12 kDa band in renal medullary samples with either one or two lower molecular weight bands, ranging from 8–10 kDa, while cortical samples consistently showed three bands of similar density, ranging from 8–12 kDa (Figure 3 and S1). A western blot of the same samples showed reactivity of the three bands with anti-canine AA antibody (Figure 3 and S2) and no reactivity with the secondary antibody only (Figure S3). These results indicate that the dominant insoluble protein in the amyloid-laden kidney was consistent with AA.

### Sequence analysis of serum amyloid A and additional insoluble proteins by mass spectrometry

To further identify the amyloidogenic protein, the insoluble protein fraction from amyloid positive (n = 7) and negative (n = 3) fox kidneys was resuspended in urea and analyzed by HPLC-ESI-MS/MS. The peptides were quantified by spectral counting, normalizing for peptide length. Peptides from SAA were the most abundant in 6 of 7 amyloid positive kidneys (13 to 48%), second most abundant in one amyloid positive kidney (6%), and absent from all amyloid negative fox kidneys (Table 3). Other amyloidogenic proteins were detected in amyloid positive and negative kidneys, including immunoglobulin light chain-λ, apolipoprotein A–I, fibrinogen-α, transthyretin, β-2 microglobulin, and lysozyme (the latter four at <1%). Only amyloid positive kidneys contained apolipoprotein A-II (1/7), apolipoprotein A-IV (Apo A-IV) (6/7), cystatin-C (2/7), and gelsolin (4/7). Compared to SAA, the contribution of these proteins was minimal, confirming SAA as the dominant amyloidogenic protein in amyloid-laden kidneys (Table 3).

**Table 3:** Percent of total insoluble protein composed of amyloidogenic proteins in renal cortex and medulla.

			% Total protein										
							Apolipoproteins						
	Animal ID	Kidney region	AA	Ig-λ	Fib-α	TTR	A-I	A-II	A-IV	β2M	Cys	Gel	Lys
	5	cortex	**42.32**	2.50	0.57	0.05	0.22	0*	0.13	0	0	0	0
		medulla	**39.42**	2.28	0.21	0	0.12	0	0.48	0	0	0	0.33
	7	cortex	**0**	0.90	0.21	0	0.08	0	0	0.02	0	0	0
		medulla	**24.17**	2.99	0.16	0.17	1.06	0	0.95	0.08	0	0.03	0
**Amyloid**	6	cortex	**26.31**	4.00	0.39	0.08	1.76	0	0.01	0	0	0	0.04
**positive**		medulla	**12.59**	2.60	0.55	0	2.59	0	0	0	0	0	0
	8	cortex	**0**	5.55	0.10	0.02	0.29	0	0	0	0	0	0.10
		medulla	**48.31**	1.91	0.08	0.03	0.42	0	0.33	0	0	0.04	0.10
	4	cortex	**5.57**	1.96	0.05	0	0.21	0	0	0	0	0	0.04
	9	medulla	**19.74**	3.12	0.14	0.57	2.08	0.10	0.71	0	0.14	0.07	0
	10	medulla	**33.21**	2.98	0.95	0.08	1.73	0	0.51	0	0.26	0.02	0
	1	cortex	**0**	4.10	0.03	0	0.52	0	0	0	0	0	0
		medulla	**0**	4.41	0.10	0	0.68	0	0	0	0	0	0
**Amyloid**	2	cortex	**0**	1.47	0	0	0.28	0	0	0	0	0	0.21
**negative**		medulla	**0**	3.11	0	0	0.40	0	0	0.02	0	0	0
	3	cortex	**0**	3.37	0.03	0.42	0.71	0	0	0.01	0	0	0.09

Ig = immunoglobulin; Fib = fibrinogen; TTR = transthyretin; β2M = β2 microglobulin; Cyr = cystatin C; Gel = gelsolin; Lys = lysozyme. * 0 = normalized spectral count of <0.01%.

**Figure 4:**
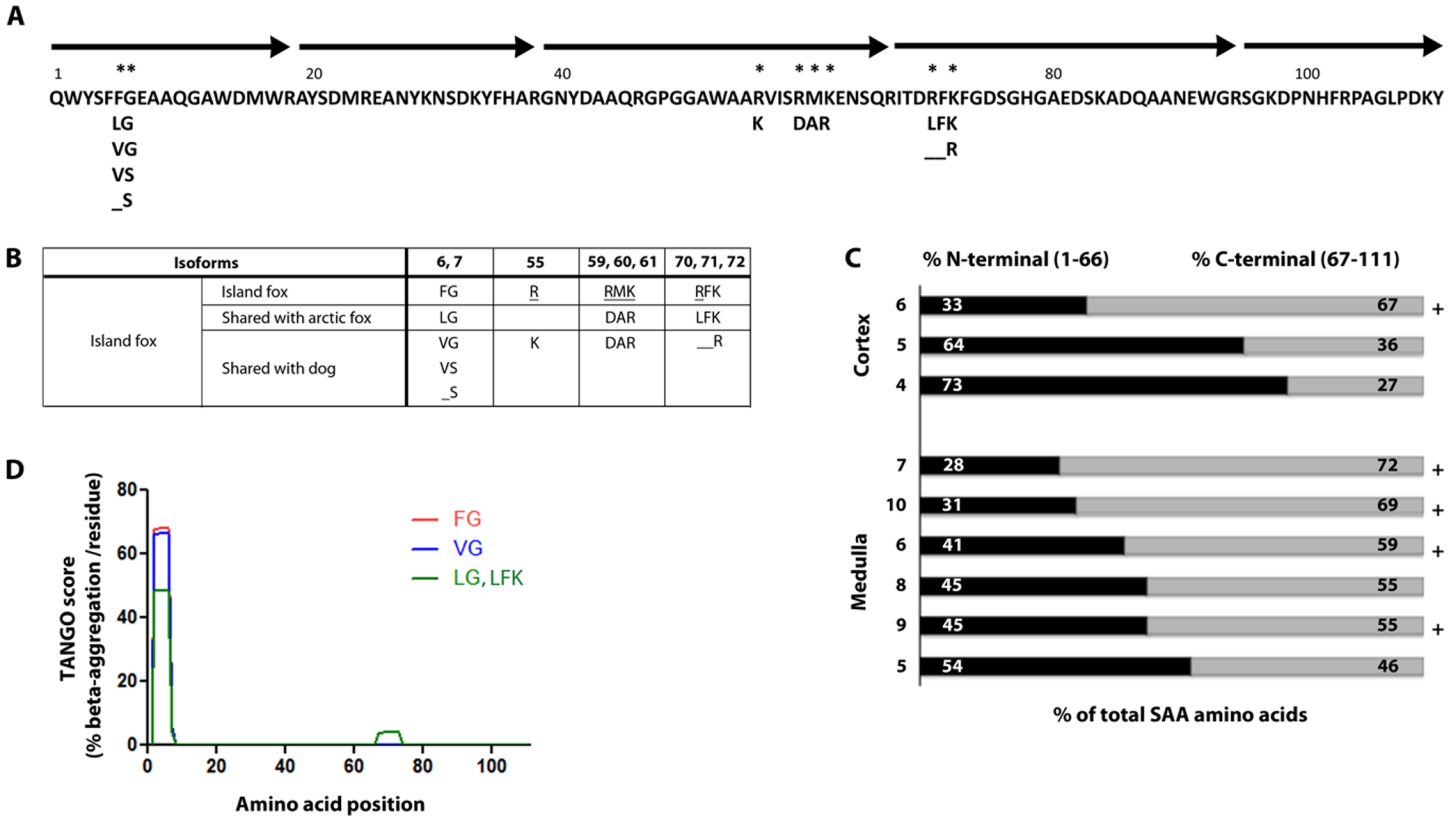
Island fox SAA protein sequence and relative abundance of N- and C-terminal peptides. **A:** The island fox SAA protein, reassembled from tryptic fragments (black arrows), is composed of 111 amino acids. **A, B:** Island fox SAA protein has 8 sites of amino acid variation at positions 6, 7, 55, 59, 60, 61, 70, and 72 (*). Some isoforms are shared with domestic dog and arctic fox; however five amino acid differences are unique to island fox amongst carnivores (_). **C:** The contribution of N-terminal and C-terminal peptides to insoluble SAA varies in renal cortex and medulla. Peptides that are within 2 amino acids of the C-terminus contribute to insoluble amyloid in 5 of 9 samples (+). Animal identifiers correspond to animals in Figure 4. **D.** Prediction of amyloidogenic regions of island fox SAA polymorphisms using the algorithm Tango [15].

To establish the sequence of SAA, overlapping tryptic peptide fragments were aligned and revealed full length SAA. Island fox SAA was composed of 111 amino acids; segment 32–44 was identical to other mammals (Figure 4). Fox SAA contained the 8 amino acid insert (residue 70–77) present in most mammals except human, mouse, and hamster (Figure 4A). Island fox SAA was similar to dog and arctic fox SAA and shares isoforms reported in these species. There were 8 sites with amino acid variation and five variants were unique to island fox among carnivores. Unique isoforms included R at position 55, RMK together at position 59–61, and RFK together at positions 70, 71, 72, (Figure 4A, B). Due to the likely homology between genes SAA1 and SAA2, peptides contributing to the full length SAA protein may be products of one or more SAA genes.

We next compared the contribution of N-terminal (amino acids 1–66) and C-terminal (amino acid 67 to 111) peptides to the total SAA peptide count. While the proportion of N-terminal and C-terminal peptides varied between individuals and between cortical and medullary samples, both N- and C-terminal peptides were detected, and there were always peptides within 18 amino acids of the C-terminus (Figure 4C). There was notably 100% homology with the C-terminal 77–103 consensus sequence, including the four basic residues implicated in binding to heparan sulfate [Arg86, Lys89, Arg95, Lys102] [43].

**Figure 5:**
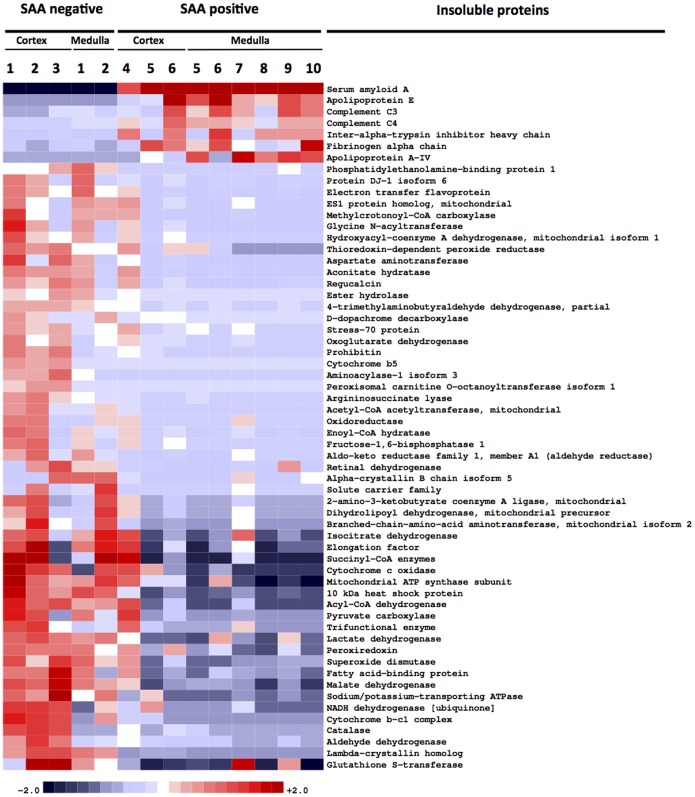
Protein abundance in amyloid and non-amyloid cases. Mass spectrometry analysis reveals SAA as the dominant insoluble protein, only in cases with histologic amyloid. Amyloid-associated proteins apolipoprotein E and A-IV were significantly increased by 1.6 and 1.3-fold, respectively, in cases of amyloidosis, as were complement C3 and C4, fibrinogen, and inter-α-trypsin inhibitor heavy chain.

An analysis of differential protein abundance revealed apolipoprotein E (Apo E) exclusively in kidneys containing SAA (7/7), consistent with common amyloid-associated proteins in humans [27], [44], [45] (Table 3). Apo A-IV was present in most kidneys containing SAA (6/7) and at very low abundance (0.007%) in one sample without SAA. Thus Apo E and Apo A-IV in SAA positive samples were at significantly elevated levels, 1.6- and 1.3-fold, respectively, as compared to controls, (FDR ≤0.05) (Figure 5). We did not identify serum amyloid P (SAP), which commonly co-exists with amyloid, possibly due to poor sequence similarity with human and mouse SAP sequences added to our dog database (the dog SAP sequence is unknown). In SAA positive samples, there were also significant increases in fibrinogen-α chain, complement C3 and C4, and inter-α-trypsin inhibitor heavy chain, which binds C3 and C4 [46]. Mitochondrial enzymes were reduced in SAA positive samples.

### Amyloidogenicity of island fox SAA isoforms

Based on the presence of SAA isoforms in the island fox, we lastly assessed the amyloidogenic potential of these polymorphisms using three different prediction programs: Tango [15], Waltz [37], and Zyggregator [38] (Figure 4D). Intriguingly, the programs were in agreement on two points, (1) amino acids 2 to 7 comprise the most amyloidogenic segment, consistent with published findings that human residues 2–8 are predicted to be amyloidogenic [10], and (2) at position 6 and 7, three of four island fox isoforms (all except VS) had high predicted aggregation potential. The FG isoform had the highest aggregation propensity. Interestingly, mink and cattle also have an FG at positions 6 and 7, but in cattle, the flanking residues are predicted to decrease the overall amyloidogenicity.

## Discussion

Although chronic inflammatory states and elevated SAA levels are common to many diseases, AA amyloidosis occurs only in select individuals, and the molecular and genetic underpinnings of susceptibility are poorly understood. Here we report systemic AA amyloidosis at a high prevalence of 34% in necropsied island foxes. In the island fox, three of four SAA isoforms, Phe6-Gly7, Val6-Gly7, Leu6-Gly7, are predicted to have higher protein aggregation propensity as compared to Val6-Ser7, which could increase their risk for developing AA amyloidosis. In contrast to the island fox, no cases of AA amyloidosis have been reported in the closest genetic relative, the mainland gray fox, which is also exposed to bacteria, viruses and parasites [47]–[51]. Since the small, isolated population of island foxes has experienced a series of bottlenecks limiting their genetic diversity [26], pathogenic mutations that increase the amyloidogenicity of the SAA protein or aberrantly elevate SAA transcription may have emerged. Taken together, a genetic mutation that alters the SAA protein sequence, expression levels, or inflammatory response may underlie the high prevalence of AA amyloidosis.

In addition to a potential genetic cause, it is also possible that AA amyloid could spread among individuals through oral exposure to AA seeds, similar to the horizontal spread of prions [52]. Transmission of AA amyloidosis has been shown in mouse models following intravenous injection of murine AA fibrils coupled with an inflammatory stimulus [53]. Similarly, chicken AA amyloidosis was also transmissible following oral inoculation with avian AA fibrils and concurrent vaccination [54]. Captive cheetahs have an extraordinarily high prevalence of AA amyloidosis (70%) and were shown to shed AA fibrils in feces, and these fibrils seeded AA amyloid in mice [55]. These results led to questions of AA naturally spreading as a transmissible amyloid [56]. Thus foxes with AA amyloidosis could conceivably contaminate the environment with AA fibrils shed in saliva, feces, or urine, particularly considering that amyloid was common in the oral cavity, intestinal tract, and kidney. Since these foxes are geographically restricted to small islands, AA fibril levels may accumulate in commonly used dens. The possibility of environmental contamination is especially intriguing in light of the 2.2-fold higher proportion of captive island foxes with AA amyloidosis (59%) compared to their free-ranging counterparts (27%). Future studies to detect AA fibrils in secreta/excreta of foxes or the soil would add to our understanding of possible transmission of AA amyloidosis in nature.

Foxes showed a high proportion of AA amyloid in organs not commonly reported in other species. Although kidney, spleen, and liver commonly accumulate AA amyloid [57]–[60], few island foxes had hepatic amyloid. Instead, a high proportion of island foxes had oral AA amyloid and some had dermal amyloid, sites more commonly associated with AL amyloidosis [61]–[65]. Island fox AA amyloid deposited heavily along basement membranes and in blood vessel walls in all organs, as seen with other amyloidogenic proteins [66] and most foxes had nearly full length AA containing the C-terminal heparan sulfate binding sites [12], [43]. Electrostatic interactions between AA and GAGs, specifically heparan sulfate proteoglycans on basement membranes, may promote aggregation [12] and widespread SAA fibril deposition through scaffolding polymerization [13], [67], [68].

SAA peptides in kidney were abundant and nearly always occurred together with high levels of Apo E and Apo A-IV, consistent with amyloid-associated proteins identified in humans [27], [44]. Additional acute phase proteins, fibrinogen-α chain and complement C3 and C4, along with a C3/C4 binding protein, inter-α-trypsin inhibitor heavy chain, were also significantly elevated. This may reflect (1) a passive co-localization of apolipoproteins and acute phase proteins with AA amyloid [69], or (2) an active role for these proteins in amyloid aggregation. Apo E3 was shown to promote AA fibril formation in vitro [70] and in mice [71], although apo E deficient mice can develop AA amyloidosis [71], [72]. The Apo E4 variant is a major genetic risk factor for late onset Alzheimer’s disease [73]. Further studies on the role of apo E in the pathogenesis of AA amyloidosis are warranted.

The isolation of the island fox on small islands together with the unique SAA protein sequence suggest a genetic cause for fox amyloidosis, however, natural transmission of AA aggregates through direct contact or environmental exposure should also be considered as potentially causal.

## Supporting Information

Figure S1 **Coomassie blue stain of insoluble proteins in amyloid-laden kidney.** Coomassie blue stain of insoluble proteins from the renal cortex and medulla show dominant bands at 12, 10 and 8 kDa. Distinct protein bands at approximately 170 kDa in three of five positive samples and at approximately 70 and 50 kDa in one positive sample do not consistently react with anti-canine AA antibody (Figure S2) and show immunoreactivity with the secondary only (Figure S3).
(TIF)

Figure S2 **Western blot of insoluble proteins in amyloid-laden kidney.** An immunoblot shows that the 12 kDa and lower molecular weight bands react with anti-canine AA antibody. In positive samples, there are immunoreactive bands at approximately 20 and 35 kDa, possibly from dimers and trimers of AA.
(TIF)

Figure S3 **Western blot of insoluble proteins in amyloid-laden kidney and spleen immunolabelled with secondary antibody.** An immunoblot shows that goat anti-rabbit HRP does not immunoreact with low molecular weight bands corresponding to SAA. Higher molecular weight bands between approximately 70 and 150 kDa appear in longer exposures of the membrane (11 minutes) and with application of an ultrasensitive substrate (SuperSignal West Femto Chemiluminescent Substrate, Pierce).
(TIF)
