# Supplementary figures and images for: Proteomic Analysis of Highly Prevalent Amyloid A Amyloidosis Endemic to Endangered Island Foxes
Source: PLoS One. 2014 Nov 26;9(11):e113765. doi: 10.1371/journal.pone.0113765 (PMC4245998; doi:10.1371/journal.pone.0113765)

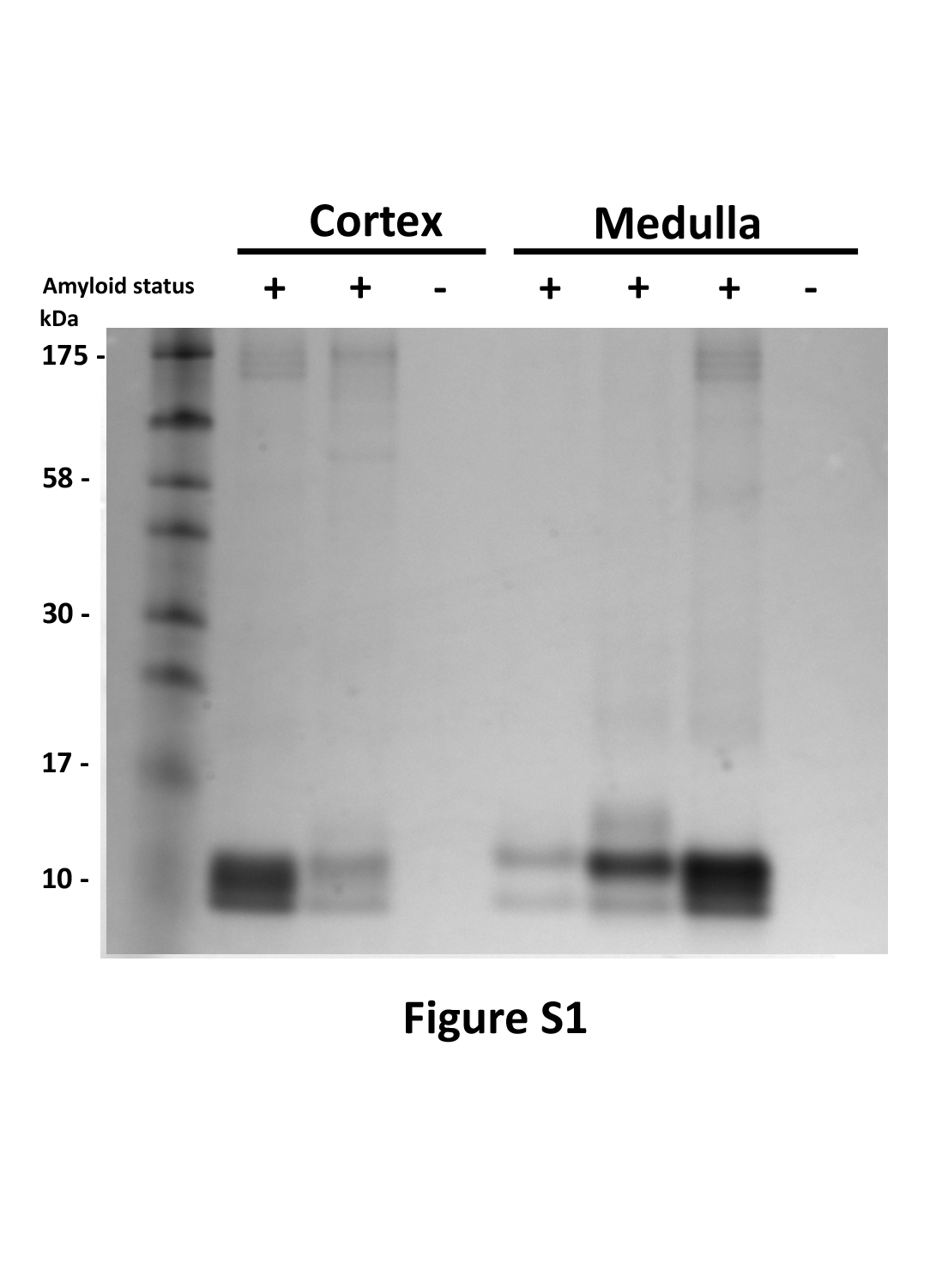

Supplement: Figure S1 — Coomassie blue stain of insoluble proteins in amyloid-laden kidney. Coomassie blue stain of insoluble proteins from the renal cortex and medulla show dominant bands at 12, 10 and 8 kDa. Distinct protein bands at approximately 170 kDa in three of five positive samples and at approximately 70 and 50 kDa in one positive sample do not consistently react with anti-canine AA antibody (Figure S2) and show immunoreactivity with the secondary only (Figure S3). (TIF) [file pone.0113765.s001.tif]

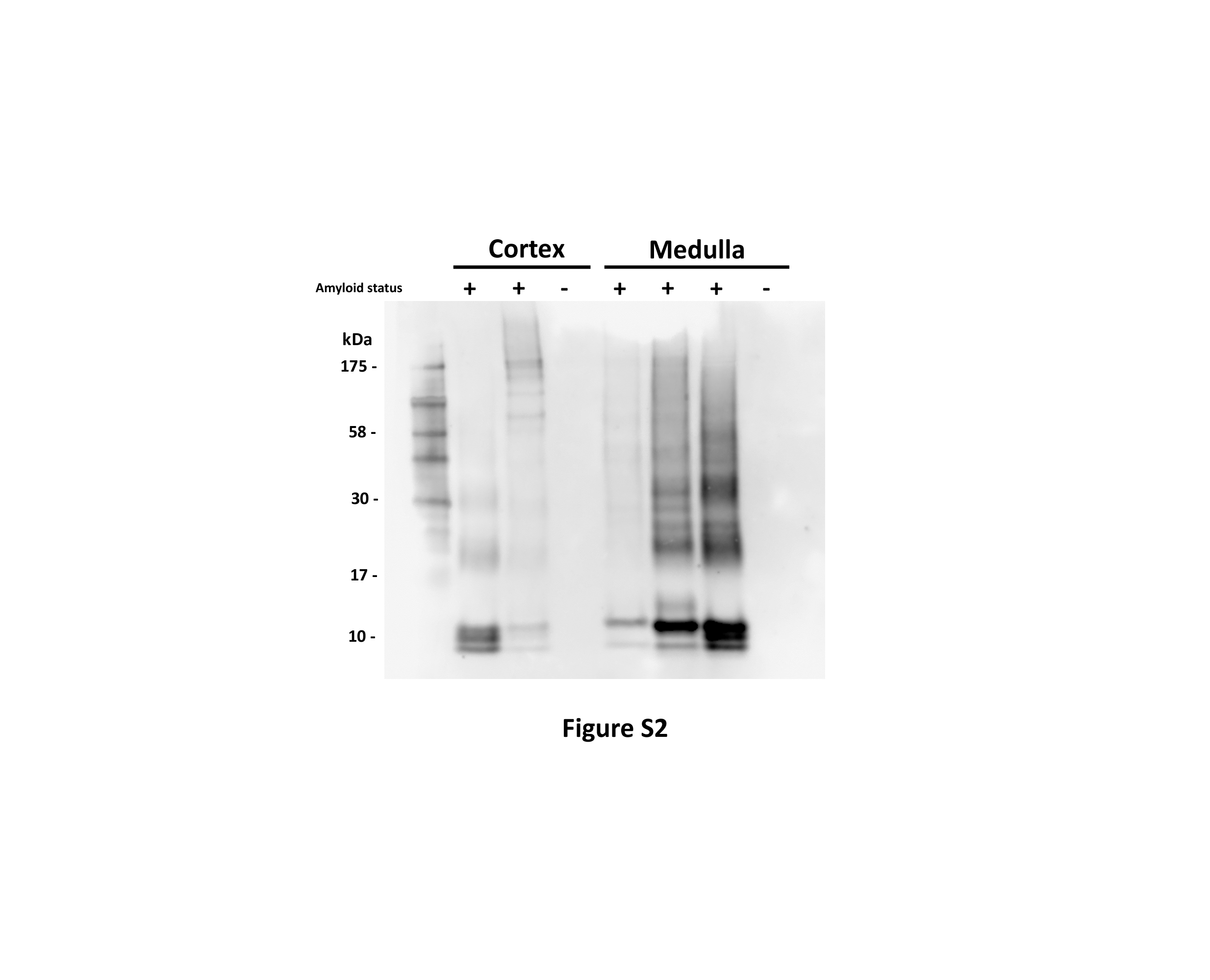

Supplement: Figure S2 — Western blot of insoluble proteins in amyloid-laden kidney. An immunoblot shows that the 12 kDa and lower molecular weight bands react with anti-canine AA antibody. In positive samples, there are immunoreactive bands at approximately 20 and 35 kDa, possibly from dimers and trimers of AA. (TIF) [file pone.0113765.s002.tif]

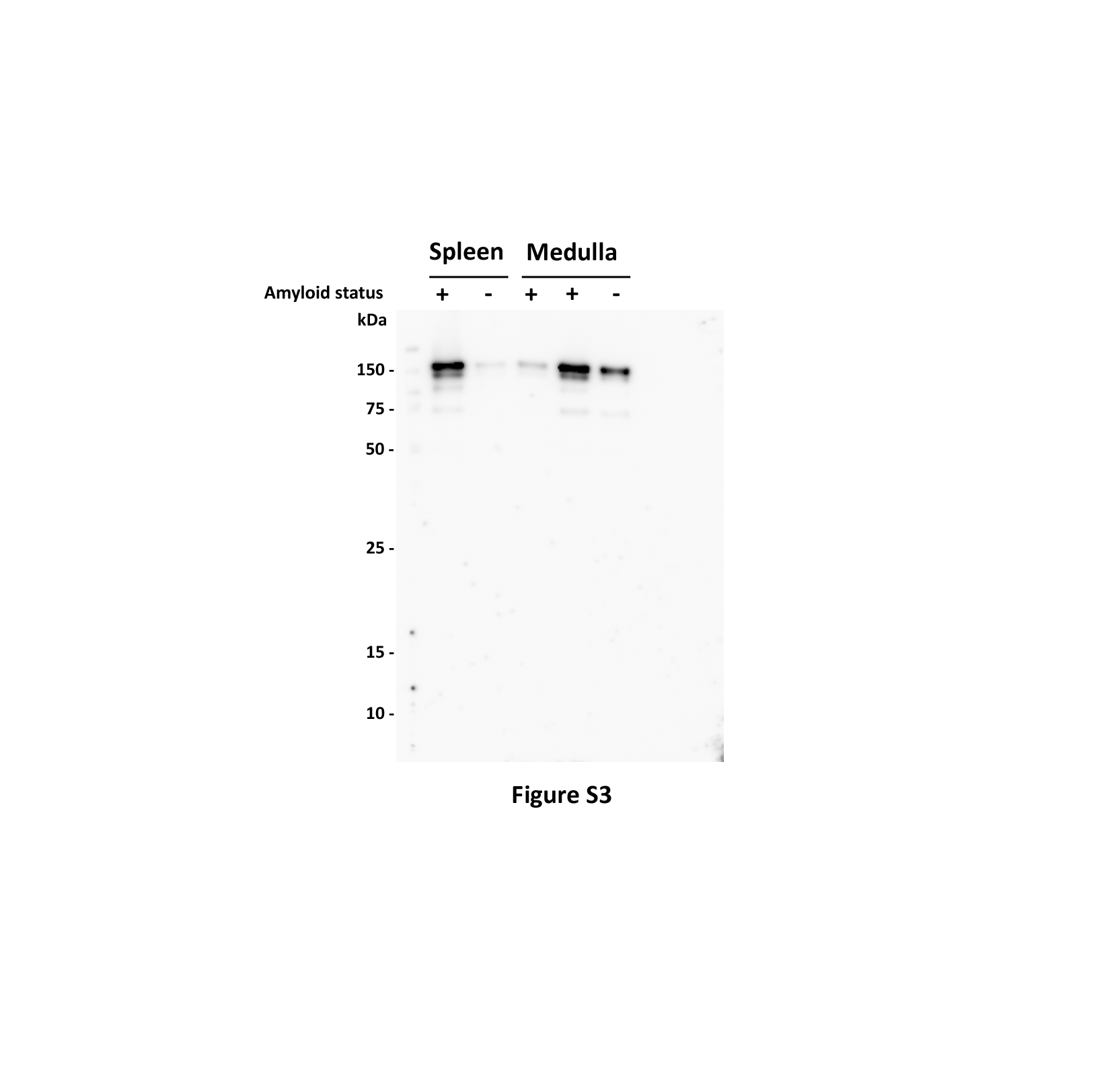

Supplement: Figure S3 — Western blot of insoluble proteins in amyloid-laden kidney and spleen immunolabelled with secondary antibody. An immunoblot shows that goat anti-rabbit HRP does not immunoreact with low molecular weight bands corresponding to SAA. Higher molecular weight bands between approximately 70 and 150 kDa appear in longer exposures of the membrane (11 minutes) and with application of an ultrasensitive substrate (SuperSignal West Femto Chemiluminescent Substrate, Pierce). (TIF) [file pone.0113765.s003.tif]
